# Patient experiences of the Long COVID–Optimal Health Programme: a qualitative interview study in community settings

**DOI:** 10.3399/BJGPO.2023.0137

**Published:** 2024-02-21

**Authors:** Hiyam Al-Jabr, Karen Windle, Andrew Clifton, David R Thompson, David J Castle, Chantal F Ski

**Affiliations:** 1 Primary Community and Social Care, University of Keele, Keele, UK; 2 Midlands Partnership University NHS Foundation Trust, St Georges Hospital, Stafford, UK; 3 Centre for Applied Dementia Studies, University of Bradford, Bradford, UK; 4 School of Health and Sports Sciences, University of Suffolk, Ipswich, UK; 5 School of Nursing and Midwifery, Queen’s University Belfast, Belfast, UK; 6 Department of Psychiatry, University of Tasmania, Tasmania, Australia; 7 Centre for Mental Health Service Innovation, Tasmania, Australia

**Keywords:** Long COVID, post-acute COVID-19 syndrome, mental health, Optimal Health Programme, primary health care, feasibility studies, qualitative research

## Abstract

**Background:**

Long COVID (LC) symptoms persist 12 weeks or more beyond the acute infection. To date, no standardised diagnostic or treatment pathways exist. However, a holistic approach has been recommended. This study explored participants’ experiences of a Long COVID–Optimal Health Programme (LC–OHP); a psychoeducational self-efficacy programme.

**Aim:**

To explore perceptions and experiences of people with LC regarding the LC-OHP and identify suggestions to further improve the programme.

**Design & setting:**

Qualitative study with patients with LC recruited through community settings.

**Method:**

This study is part of a wider randomised controlled trial. Eligible participants were aged ≥18 years, have LC, and attended a minimum of five LC–OHP sessions plus a booster session. We interviewed those randomised to the intervention group. Interviews were conducted by an independent researcher and thematically analysed to identify common, emerging themes.

**Results:**

Eleven participants were interviewed, mostly women from a White British ethnic group (*n* = 10). Four main themes were identified, reflecting programme benefits and suggestions for improvement. The programme demonstrated potential for assisting patients in managing their LC, including physical health and mental wellbeing. Participants found the programme to be flexible and provided suggestions to adapting it for future users.

**Conclusion:**

Findings support the acceptability of the LC–OHP to people living with LC. The programme has shown several benefits in supporting physical health and mental wellbeing. Suggestions made to further adapt the programme and improve its delivery will be considered for future trials.

## How this fits in

Long Covid (LC) is a new illness with a wide range of fluctuating symptoms. There are still lots of unknowns surrounding its trajectory, and no standard care approach is yet available. In the UK, the number of people with LC is increasing and comprehensive care has been recommended to address the individual needs of those affected. This study provides insights of patients’ experiences with the Long COVID–Optimal Health Programme (LC–OHP); originally designed to support mental wellbeing and physical health. Findings highlighted the benefits of the programme and tangible ways to improve delivery. In addition, recognising the heterogenous pleomorphic nature of LC, participants emphasised the importance of communication skills by practitioners as well as the implementation of personalised care that could meet the needs of individual patients.

## Introduction

The COVID-19 pandemic has resulted in more than 767 million confirmed cases and six million deaths globally.^1^ For most, the acute COVID-19 infection resolves within 2–4 weeks. However, for many, long-term symptoms persist for weeks or months or even years, with a set of symptoms that has been labelled ‘long COVID’ (LC). LC has caused unprecedented burden on health services.^2–6^

LC is described as an illness with persistent symptoms or where new symptoms continue to appear beyond the 2–4 weeks of acute infection, and where these symptoms cannot be explained by or attributed to alternative conditions.^7,8^ It is a multisystem disease with a possible 200+ symptoms that impact on people’s health, affecting their daily activities.^9–16^ LC has been associated with deteriorating mental health^11,17–19^ and increased risk of neurological conditions.^20^ LC risk factors include female sex, older age, low socioeconomic status, concomitant chronic illness, obesity, smoking, and initial disease severity.^2–4,21–25^

To date, no standardised approach exists for diagnosis or management of LC.^26–29^ Given the longitudinal nature and multiplicity of LC symptoms, patients need appropriate support to manage their condition,^30,31^ much of which is unavailable (for example, psychological support, and self-management practices).^29^ In light of LC being a relatively new condition, few studies have reported on the LC experience from the perspective of the patient. Of those who have, less favourable experiences have been reported with the current support systems, including poor recognition of their condition.^32^ This leads many to seek alternative support, including switching to social media platforms for advice.^29^ However, this could pose further challenges as experiences with LC are different and these platforms are potentially a source of conflicting information.^33,34^ Additionally, many people reported taking over-the-counter products, supplements, and making dietary changes to manage their LC symptoms. However, there is also an associated risk with this self-prescription approach, including drug–drug interactions, and taking inadequate products.^35^ Therefore, LC demands a focus, not on symptom-by-symptom management, but on providing holistic, integrated care,^36–38^ which guides people and improves their self-management skills.

The Optimal Health Programme (OHP) is a psychoeducational programme that enhances self-management and self-efficacy through skill-building and education, to improve the physical and mental wellbeing of people with chronic medical conditions.^39–43^ It employs a holistic, person-centred approach based on collaboration and healthcare integration.^41^ It has previously effectively supported the mental health of people with long-term medical conditions, including diabetes and chronic kidney disease.^40,44,45^ However, as LC does not fit into a specific diagnostic category, the Long COVID–Optimal Health Programme (LC–OHP) was adapted from the existing OHP and tested within a feasibility randomised controlled trial (RCT).^46,47^ This study aimed to explore the feasibility and acceptability of delivering the LC–OHP to people with LC, and their perceptions and experiences with using it. The objectives were as follows: to identify views on the LC–OHP; to explore the potential for the programme to support mental and physical wellbeing; and to gain suggestions for improving the programme.

## Method

### Study design

This exploratory study is part of a wider RCT^46^ that aimed to identify the feasibility and acceptability of using the LC–OHP. The present study involved semi-structured interviews with participants randomised to the intervention arm of the RCT. The study was conducted at a local university in the UK, between December 2021 and May 2023. This study is reported according to the Standards for Reporting Qualitative Research set by O’Brien *et al*.^48^

The study was overseen by an independent data management committee that included two public members with LC experience. The committee met four times virtually over the course of the study and provided advice and guidance on its data management. The committee was updated at each meeting on study progress, adherence to protocol, amendments, and preliminary findings.

### Participants

Inclusion criteria were people with a confirmed diagnosis of COVID-19 infection with ongoing symptoms for LC, aged ≥18 years, who were able to understand English language, and who had attended all sessions of the programme.

The wider feasibility trial recruited a total of 60 participants, of whom 28 were randomised to the intervention group and offered the LC–OHP. This presented study aimed to interview 12–15 participants from the intervention group, dependent on data saturation.

### Recruitment

For the wider trial, participants were recruited using multiple approaches, including referral from LC clinics, through social media, and word-of-mouth. Those interested in taking part were sent a study information leaflet and consent form that also contained information about potential for participating in an interview at the end of the study. After completing the final follow-up questionnaire for the wider trial, participants in the intervention group were invited by the researcher (via email) to the final interview. Interviews were arranged at their convenience. Data collection was conducted between August 2022 and April 2023.

### Intervention

The LC–OHP is a five-session programme, delivered over at least 5 weeks (approximately 1 hour per week). Following randomisation to the intervention group, participants received a hard and an online copy of the programme. The LC–OHP was delivered virtually (for example, by Microsoft Teams) or by telephone at a convenient time. Participants could choose to receive these sessions on a one-to-one basis or in a group. To monitor participant progress and reinforce learnings, an additional booster session was delivered 3 months after the fifth session. A summary of sessions is shown in Table 1.

**Table 1.** Description of LC–OHP programme sessions

Session	Title	Content
1	Optimal health	What is optimal health? Optimal health wheel
2	I-Can-Do-Model	Strengths and vulnerabilities Stressors and strategies Health plans 1 and 2
3	Factors of wellbeing	Medication and metabolic monitoring Collaborative partners and strategies Health plan 3
4	Visioning and goal setting	Defining change Orientation and preparation Creative problem solving and goal setting Reflection and celebration
5	Building health plans	Health plans 1, 2, and 3 My Health Journal
Booster	Reflecting on the learning in the transformational journey to sustain wellbeing	Reflecting on the learning in the transformational journey to sustain wellbeing

### Data collection and analysis

An interview topic guide (Table 2) was developed by the research team and interviews were audio-recorded for analysis. The topic guide was revised after interviews and additional questions (prompts) were added where necessary, following discussion among researchers. All interviews were conducted by an independent researcher (KW, AC). Interviews were conducted via telephone or virtually as preferred by participants. Interviews were transcribed verbatim and thematically analysed (using an inductive approach) to identify common themes.^49^ Transcripts were continuously revisited, and accuracy verified by listening to recordings and comparing these with the transcripts. Coding of data was conducted using Atlas.ti-23 software. Codes and themes were reviewed by other members of the research team (KW, AC) to establish rigour and ensure consistency. Analysis included reading each transcript and creating an initial list of individual codes. Codes with apparent relationships were combined into themes. Once initial themes were generated, they were discussed, reviewed, and refined among researchers to ensure that underlying codes of each theme formed a coherent pattern. At this stage, some initial themes were combined, and others were broken down into separate themes. Final themes were then defined and were given an appropriate label. Disagreements were resolved by consensus and by referring to the transcripts and original recordings. Interviews were carried out until reaching data saturation.^50^ Researchers met continuously to discuss the findings of each interview and agreed on reaching data saturation, which was considered achieved when no new themes or sub-themes emerged in two consecutive interviews.

**Table 2.** Semi-structured interview guide

1.Can you please tell me a little bit about why you agreed to take part in the study? 2. Describe your experience of being involved in the programme - Views on the programme (for example, did you feel listened to, respected?) - Perceived benefits - Any negative aspects or barriers encountered?
3. What do you think about the materials and support provided throughout the programme? - Booklet and sessions’ content and discussion - Programme’s activities - Materials’ relevance or suitability to long COVID - Preference to use soft or hard copy of the booklet.
4. Do you feel that there may be changes that could be made to the programme? What, how, why? - Anything that could have been done differently in relation to the programme? - Thoughts about using the programme in the future - Programme’s fit within care provided to patients with long COVID - If this were to be carried out by the NHS, who do you think might be involved in delivering the programme? - Is there anything you feel we should have talked about and haven’t? Anything else you would like to share?

## Results

Table 3 shows demographics of recruited participants. Eleven participants were interviewed. Ten interviews were conducted virtually, and one by telephone. Interviews lasted an average of 21 minutes (range 13–31 minutes).

**Table 3.** Participant demographics and recruitment strategies (*n* = 11)

Characteristic	Frequency, *n*
**Age, years**	
18–29 30–39 40–49 50–59 ≥60	1 3 4 2 1
**Sex**	
Female Male	10 1
**Ethnic group**	
White British Other White backgrounds	10 1
**Education**	
Post-secondary education Undergraduate degree Post-graduate degree Vocational qualification	2 3 4 2
**Recruitment method**	
Referral from a LC clinic Social media Research studies website Other LC support groups	2 3 3 3

LC = long COVID.

All participants received the programme on a one-to-one basis by the same researcher (HA). All participants completed all sessions (range 6–9 sessions in total).

Participants were aged between 26 years and 65 years, from various professional backgrounds including healthcare professionals (HCPs) and social workers. Ten participants were female. Duration of their LC ranged between 4 months and 25 months (median 9 months).

### Semi-structured interviews

Four overarching themes emerged. These are described below with supportive, anonymous quotes. To further protect participants’ anonymity, the quotes are coded with new ID codes other than the ones used during the study.

### Theme 1: Benefits of the LC–OHP

The LC–OHP was well-received by participants who reported benefits in supporting them to self-manage their LC. This encompassed enhanced communication and reaching out to others for assistance; positive thinking strategies; planning to improve coping; self-exploration and acceptance; social inclusion; feeling empowered; and validating their experiences. The programme also helped participants to approach LC-induced challenges differently and to make adjustments to better manage their life alongside LC.

*‘I mean in terms of me, it really did help focus my mindset and think about things in a new way and actually communicate to others how I’m dealing with things and get help and support and without that, I think I’d still be stuck on waiting lists struggling with things.’* (Participant-H)

Participants agreed the LC–OHP helped them to shift their thinking around health-related goals, and not to wholly focus on the negative aspects of their illness. It also helped them establish coping mechanisms and self-management tools; for example, through forward planning of activities. Some perceived it as an opportunity to be ‘actively’ heard, enabling them to express their feelings and not to feel alone or judged. For some, this was the only time they felt listened to and their feelings acknowledged

*‘I found it probably one of the most helpful interventions and methods of support that I’ve had throughout my whole Covid experience, it’s been definitely the most useful and it helped me in … sometimes when I’ve been feeling pretty down about it all,’* (Participant-F)

Participants reported that the LC–OHP resulted in a better understanding of their illnesses, insightful reflection, and self-acceptance. Participants identified that the LC-OHP tools helped them regain a level of control over their lives.

Some participants, while experiencing long waits for LC-related health appointments, had been proactive in self-management, engaging with a range of resources to build their knowledge of LC, and to navigate through LC. All found that the programme focus validated these actions.

Despite advantages of the LC–OHP, some encountered challenges, including accessing and interacting with an online copy of the programme, and completing its associated activities. They felt this could be onerous, especially with LC fatigue and brain fog, to document and discuss daily activities. Although no participant was pressured to complete the activities, some did push themselves to get the best out of the programme.

Other challenges included lack of clarity on certain sections of the programme, not sufficiently capturing neurological and cognitive aspects of LC, and needing more time in some sessions for discussion. Some also added that certain sessions felt heavy to be completed in one encounter:

*‘The only thing is a more like suggestions …. One thing I did write was I thought the stress part was really interesting, but we went through it a bit fast, I thought maybe there could be more emphasis on that.’* (Participant-D)

Additionally, one participant described some parts of the programme booklet as requiring someone else to assist them to get a better understanding, especially when feeling fatigued. A few other participants talked about challenges related to the information technology when receiving sessions, such as interruptions to internet connections.

### Theme 2: Programme materials, delivery logistics, and relevance to long COVID

Programme content and activities were valued by all participants, with many commenting on the relevance of sessions to their situation. Participants found that the components of the LC–OHP were well-structured, addressed most LC symptoms, and could be customised to individual needs:

*‘I think the sessions were perfectly suitable to LC because the more I’ve had this condition it feels like a long-term condition which I’m presuming that programme is used for, and there are all sorts of aspects to … I mean I understand there’s mental health element of it, is sort of encompasses every part of your psychological health … all aspects of your life really.’* (Participant-I)

The programme was described as clear, easy to understand, well-written, colourful, and with a good use of visual aids that conveyed key messages without reading lots of text. As nearly all participants experienced LC-induced ‘brain fog’, this format made it easy for them to process the information.

Participants described elements of the LC–OHP as particularly useful, including those that assisted them to find a balance in their daily routines by planning and monitoring their activities and energy levels, helping them acknowledge achievements, and identifying people in their network who could offer support. Many reported the holistic approach made the programme applicable, not only to LC, but also to other aspects of their lives.

Most participants reported that the sessions were delivered at the right time with good timing of breaks. They described sessions as running smoothly and valued that they were adjusted according to individual needs. However, some participants argued that the best time to deliver the programme would be early in the LC journey to ensure the delivery of essential information and/or activities to initiate prompt understanding and self-management.

Participants also spoke of receiving hard and soft copies of the LC–OHP. The former was considered useful for people with difficulty using technology and as less energy draining than staring at a screen. Additionally, the hard copy enabled note writing, and refreshing memory of previous discussions.

Several participants perceived that remote delivery was helpful in facilitating participation. This made them feel the programme was responsive to their needs, reducing barriers to engagement.

*‘I think it worked really well,* [it was] *reassuring to do because I know some studies you have to attend in person, so I think the fact that this study is done online without being seen face to face is like massive benefit.’* (Participant-C)

### Theme 3: Suggestions for improving and future potential of the LC–OHP

Many participants expressed that no changes were needed to the programme. Others offered suggestions for improvement, including adding more writing space for programme activities, adding an additional session, and adding more detail to certain sections (for example, introduction, fight-or-flight response, stress, physical activity, and post-exertional malaise). Some suggested developing an ‘app’ for the programme to facilitate interaction:

*‘... well, I don’t know if it’s possible to have it as an app … that’s probably again something that I would interact with quite easily if it was sort of something that I could access on my phone rather than something I might actually log into computer for.’* (Participant-J)

Contrasting perceptions to peer support were seen. One participant explained that meeting other people with LC and sharing experiences could provide new learning approaches and offer additional support, while another argued that group discussions do not always work, at least not for all people. However, this participant viewed the programme to be flexible in terms of its delivery in a one-to-one basis, in groups, or even through self-direction to the programme materials.

*‘So this programme is a lot more aimed at you, rather than aimed at groups, groups don’t always work, … and it could be more online as in groups … and it can be one to one, and it can be learnt even probably solo without … with just the like the … the information, you could teach it yourself so most people would be able to go through that booklet and gain something from it without needing to speak to somebody.’* (Participant-A)

Regarding future use of the programme, most expressed willingness to using it again. Some reported incorporating many of the programme’s tools into their daily activities and work.

*‘I think I learnt a lot on the programme that I’m taking forward you know, it’s like I’ve changed all the things I do in response to going through the programme, such as managing my activities better now, I think I’m pacing a lot more effectively for example, so I think those are lessons I take forward anyway.’* (Participant-D)

When asked who might be involved in delivering the programme, some suggested it should be delivered by professionals, such as counsellors, physiotherapists, dieticians, psychologists, and through existing LC services or clinics. One participant felt that it could be provided through GP practices to help them support people with LC, especially in the absence of clear management approaches. Two others suggested it did not need a HCP to deliver it; with proper training it could be delivered by most people with a health background. Alternatively, it could be delivered by patients themselves, with appropriate support by a practitioner. This was noted as potentially helping to ease the pressure on medical staff and to further engage and upskill those with LC.

### Theme 4: Other long COVID supports

Although exploring other support for LC was not a specific aim of this study, some shared other resources they had experienced. These included support from different HCPs, work, family and friends, and other LC support programmes.

Participants had mixed experiences of practitioner support. Some reported positive relationships and mentioned a range of different practitioners involved in their care. They also mentioned being referred to other LC clinics through their GPs for further support and described the learning they gained from working with different people.

*‘and the … support I was getting through the NHS .… the LC clinic again I found really really helpful, my doctor was really good, and actually … referred me to that* [LC clinic] *and I think I started doing that certainly before I met up with* [researcher] *… they were talking more about relaxation techniques at the LC clinic that sort of thing, whereas with* [researcher] *I was thinking more about my lifestyle … we did touch on relaxation and breathing stuff but … it was sort of slightly different so I actually benefited.’* (Participant-K)

However, other participants described less positive experiences and subsequent efforts they had to take to seek additional resources.

*‘I was not getting anywhere with any support from my GP, from occupational health at work, I was at that point waiting for my referral for the LC hub to go through, so it was really an attempted self-help and trying to do whatever I could to get back to work and get back to normal, I figured it might be beneficial so give it a try.’* (Participant-F)

Most participants expressed encountering a lack of understanding of their LC condition from their GPs. Although this was to a certain degree accepted, recognising that LC is still a new illness with a limited evidence base and while acknowledging the busy schedule of GPs, some highlighted the importance of following a personalised approach of care and the use of effective consultation skills when interacting with patients.

*‘... so if your doctor is just signing off month after month your fit notes … it was … about the fourth month I was like wow you could at least speak to me to see how I am … that would be nice … yeah just having more personalised just a chance to speak to somebody.’* (Participant-G)

Many participants also talked about being engaged in other LC support groups provided through work or other research programmes. Talking to others with similar experiences was considered highly important and helped in supporting their wellbeing in not feeling alone in this journey and allowed exchanging of ideas.

## Discussion

### Summary

This study explored the experiences and views of people with LC who received the LC–OHP. Most participants were females aged >43 years, and from a White ethnic group. Overall, the LC–OHP was perceived as relevant and useful. Notably, the programme was reported as empowering patients by enhancing their control over symptom management. Participants reported several benefits to the programme, including shifting their thought processes to adopt a more positive outlook, improving their coping mechanisms, and prioritising activities to assist them in leading a better-planned or more manageable life. They appreciated the holistic, person-centred approach of care provided by the programme, as they found it effective for responding to the multiple needs of LC, rather than using a ‘one-size-fits-all’ approach. An important characteristic of this programme was flexibility and tailoring to the uniqueness of everyone’s journey. In this sense, the LC–OHP has potential to provide a set of tools to achieve balance between symptom management and daily activities. It allowed participants to practise self-acceptance and self-growth to make suitable adjustments to their new, altered life. Thus, the LC–OHP showed promise for supporting people with building self-efficacy and capacity.

Participants agreed the programme content was well-presented, with clear, easy-to-understand language, with colours and visual aids that facilitated learnings. It was also perceived as LC-relevant, covering the most common symptoms. They found that the LC–OHP offered coping strategies that could be adjusted to suit individual needs, helping participants with self-exploration and to identify their limits. Participants made some suggestions for improvement, including adding extra detail to certain sections.

Delivering the programme in groups was suggested to promote sharing of experiences and knowledge, and for symptom validation. The LC–OHP can be delivered in groups, although in this occasion, participants opted for one-on-one session delivery. Participants also suggested that trained HCPs or patients could deliver the LC–OHP under LC services. Mode of delivery was reported to be a facilitator for involvement. Providing flexibility with regard to time and mode of sessions was important to participants, owing to the fluctuating nature of this illness.

LC interactions with practitioners reflected a mix of experiences: while some were positive, overall, more negative experiences were described. The latter included perceived insufficient support and not being believed. Participants emphasised the need for practitioners to use effective communication skills, and to offer validation of their experiences.

### Strengths and limitations

There are several strengths to this study. First, to reduce the risk of social desirability bias, all interviews were conducted by independent researchers who were not involved in programme delivery. This allowed participants to share their experiences openly with someone they had no previous relationship with and might have contributed to collecting less biased responses. Second, to ensure consistency, most interviews were conducted by the same interviewer using a uniform topic guide. Third, several techniques were used to strengthen the rigour of the qualitative data analysis, including reading transcripts while listening to recordings to ensure everything was accurately captured, discussing emerging themes with other researchers, and referring to transcripts in case of disagreements. Fourth, the study was overseen by the data management committee, which included two members of the public with experience of LC.

The study also has limitations. Most participants interviewed were females from a White British ethnic group. Therefore, further research is needed to identify the impact of the LC–OHP on males, and those with other ethnic backgrounds. Another limitation was delivery of the programme on a one-to-one basis. Participants’ experiences may have been different if it was delivered in a group format. In consideration of the highly varied nature of LC for one individual to the next, the findings of the study may not be generalisable to the wider community. However, this study has provided useful data to inform improving the LC–OHP for broader delivery.

### Comparison with existing literature

A higher number of female participants took part in this study; this is consistent with other studies, where female sex has been cited as a risk for developing LC.^51–58^ Other reported risk factors included older age and comorbidities.^59–65^ This is also consistent with the findings of this study, with the average age of participants >43.5 years, and most aged between 35 years and 49 years, which is an age range reported by the Office for National Statistics to be most affected by LC.^66^

Evidence to links between LC and ethnic group is still unclear, with some reports indicating lower rates of self-reported LC among non-White individuals, whereas other reports indicating higher rates of clinically diagnosed LC among people of South Asian and Black ethnic backgrounds.^67^ Owing to the small sample size included in this study and the lack of ethnic variations, further research is needed to identify the prevalence of LC and acceptability of LC–OHP among various ethnic groups.

Flexibility regarding delivery modes of sessions was also highlighted. The impact of intervention delivery modes are well-established, being telephone and/or online platforms.^68^ Several advantages were identified for remote delivery including research inclusivity, extended reach, and flexibility and convenience for participants.^68^

Programme content was well-received and described as simple, visual, and colourful. This was purposeful as it is commonly requested to use clear language when communicating with public members regarding research projects.^69,70^ This is especially important for people with LC, considering the cognitive impact of brain fog and fatigue.

Suggestions to improve the programme were mostly related to adding details to particular sections; for example, the role of stress in LC. Interventions that help people better cope with stress can improve the body’s stress response and ameliorate some LC symptoms.^71^ Another suggestion was to deliver the programme in groups to gain more learning and support. It is well-established that when patients connect socially, it reduces isolation and loneliness. Furthermore, peer support is considered an essential resource for people with LC, especially during the pandemic.^72^

Participants suggested the programme could be peer led or delivered. This approach has been used with other chronic health conditions, where experience and knowledge of patients were harnessed when supporting each other.^73,74^ This method has been shown to reduce burden on medical staff and as a cost-effective approach of care that empowers patients and increases active engagement in self-management.^75^ The OHP programme has been delivered by a range of HCPs (for example, nurse, psychologist, pharmacist),^39,40,46,76^ although not yet by patients; an approach worthy of further investigation.

Communication and support from HCPs was highlighted, with many participants describing that the communication skills of practitioners did not meet their expectations. Echoing other research studies, practitioners should employ effective communication skills and engage reflexively with their patients.^77,78^ Additionally, these skills are highlighted by the General Medical Council’s standards of professional practice.^79,80^ Unfortunately, expectations of support from practitioners often fell short of expected standards. However, the structure, format, and content of the LC–OHP has potential for enhancing patient–practitioner engagement and communications.

### Implications for research and practice

Findings support the feasibility of delivering the programme to people with LC, their acceptability of receiving it, and their overall positive experiences with using it. The LC–OHP offers a flexible approach to support both physical and mental symptoms of LC. It empowers and guides participants through their recovery, building self-efficacy. Tangible ways were articulated in which the programme can be further tailored to the individual needs of those with LC, and potentially improve delivery, including delivery by trained practitioners and also by people with LC. However, such delivery methods require further exploration and assessment to determine appropriateness in the context of the LC–OHP. Additionally, future research should aim to identify strategies to widen the diversity of participants to include the more ‘hard-to-reach’ populations including those from different ethnic backgrounds.

The use of personalised care to meet the needs and demands of individual patients is critical, especially considering the heterogenous pleomorphic nature of LC. Practitioners are advised to use effective communication skills when interacting with their patients, to validate their experiences and work together in creating an individualised care plan.
